# Privacy-Preserving Framework for Multi-Institutional Medical Time-Series Analysis via Homomorphic Encryption: Design and Development Study

**DOI:** 10.2196/87306

**Published:** 2026-08-07

**Authors:** Yao Lu, Yu Tian, Tianshu Zhou, Jing Xia, Zhensong Zhao, Huiyao Sun, Kangli Liu, Jingsong Li

**Affiliations:** 1 Research Center for Scientific Data Hub, Zhejiang Lab, Hangzhou, Zhejiang, China; 2 Engineering Research Center of EMR and Intelligent Expert System, Key Laboratory for Biomedical Engineering of the Ministry of Education, College of Biomedical Engineering and Instrument Science, Zhejiang University, 38 Zheda Road, Hangzhou, Zhejiang, 310027, China, 86 13958134365; 3 School of Intelligent Science and Technology, Hangzhou Institute for Advanced Study, University of Chinese Academy of Sciences, Hangzhou, Zhejiang, China

**Keywords:** privacy-preserving learning, homomorphic encryption, secure multiparty computation, patient data privacy, medical time-series analysis, recurrent neural networks

## Abstract

**Background:**

The development of robust medical AI for knowledge discovery and decision support commonly necessitates large-scale datasets from multiple institutions. However, such data aggregation is severely constrained by privacy regulations and the inherent risk of sensitive information leakage, making it difficult to navigate the utility-privacy trade-off.

**Objective:**

We aimed to design a secure multiparty deep learning system that enables privacy-preserving modeling from distributed medical time-series data without centralizing raw information or exposing model parameters. Our goal was to achieve predictive accuracy comparable to nonsecure models while providing strong security and efficiency.

**Methods:**

We developed a framework using threshold homomorphic encryption to securely train recurrent neural networks on distributed longitudinal data. To improve the efficiency, we proposed an optimized encrypted matrix multiplication scheme, a secure ciphertext refresh protocol, and used lightweight encryption parameters and low-degree approximated activation polynomials. The system was evaluated on 4 real-world intensive care unit datasets for tasks like mortality and sepsis prediction.

**Results:**

The system demonstrated practical efficiency, requiring approximately 1 minute per training iteration for processing 125 local batches over 39 variables and 48 time steps, and scaling well with data size and participant number. Securely trained models achieved predictive performance that was comparable to, and in some cases superior to, nonsecure centralized models, highlighting their ability to learn generalizable patterns in different unseen data distributions. For example, on the PhysioNet Challenge 2012 dataset, our secure model achieved an area under the curve (AUC) of 0.8480, outperforming the nonsecure baseline AUC of 0.8404.

**Conclusions:**

This work provides a viable and efficient solution for cross-institutional, privacy-preserving analysis of longitudinal medical data. The framework successfully bridges the utility-privacy gap, facilitating safer collaborative research and enabling robust knowledge discovery and decision support while adhering to strict data protection standards.

## Introduction

### Background

The integration of massive, multiparty patient data is crucial for developing high-quality medical AI. Compared to single-site datasets, those aggregated from multiple institutions exhibit greater diversity in patient demographics, clinical practices, and health care conditions. This enables researchers to obtain sufficient samples to train robust models for specific tasks, such as studying rare diseases [1]; achieve results with higher reproducibility and generalizability [2]; accelerate the translation of research findings into clinical practice [3]; and discover novel patterns that are undetectable in any single dataset [4]. As a result, such integration significantly promotes the progress of medical data analysis and makes their applications widespread in many real-world clinical scenarios.

However, the sharing of such data is severely constrained by various privacy regulations, such as the HIPAA (Health Insurance Portability and Accountability Act) Privacy Rule in the United States and the GDPR (General Data Protection Regulation) in the European Union [5,6]. These regulations, coupled with the severe repercussions of privacy breaches—including reputational damage, substantial financial penalties, and health care fraud—make medical institutions highly reluctant to share their sensitive patient data [7]. Consequently, there is an urgent need for secure multiparty medical AI analysis protocols that can navigate the nontrivial trade-off between practical utility and robust security.

### Prior Work

Most existing privacy-preserving AI protocols are designed for, or evaluated on, cross-sectional data. This paper, in contrast, addresses the challenge of analyzing longitudinal patient data using recurrent neural networks (RNNs) in a privacy-preserving manner. Longitudinal patient data, which records patient visits, test results, and treatments over time, provides more invaluable insights into the relationship between temporal patterns of features and events of interest [8]. RNNs are particularly well-suited for mining such data due to their ability to model nonlinear relationships and high-dimensional temporal dependencies. They have proven essential in advancing personalized medicine through disease progression modeling [9], outcome prediction [10], and treatment recommendation [11]. Despite their value, longitudinal datasets are often harder to collect and tend to be smaller in scale than their cross-sectional counterparts. This scarcity frequently necessitates pooling data from multiple providers to train robust models, a process thwarted by the distributed and sensitive nature of the data itself.

Nowadays, numerous privacy-preserving deep learning solutions have been proposed for architectures like multilayer perceptrons [12], convolutional networks [13], and generative adversarial networks [14]. However, few have focused on secure RNN-based protocols. Among them, many used federated learning and have been applied in various tasks [15-18], allowing parties to share only local intermediate results like gradients, thus enabling the training of predictive models without direct original data exposure [19]. However, several studies have shown that such aggregated information can still reveal parties’ private data [20,21], leading to privacy compromise risks even with large batch sizes and noisy gradients [22-25], significantly hindering the adoption of these solutions. Furthermore, some recent works have also found that RNN-based algorithms are more vulnerable to attacks targeting aggregated information (eg, membership inference attack) and less well-protected with differential privacy than other common neural networks, making the privacy-utility trade-off even more challenging [20,26,27].

In contrast to federated learning, cryptographic techniques like secure multiparty computation (SMPC) offer stronger cryptographic security guarantees. However, SMPC frameworks often incur substantial communication and computational overhead that escalates with the number of participating parties. This scalability challenge remains a critical bottleneck for large-scale applications. Despite ongoing optimizations, most SMPC-based RNN implementations are still confined to 2-party or few-party settings [28,29], which is often insufficient for multi-institutional medical collaborations.

Homomorphic encryption (HE) enables computation on encrypted data, making it a naturally appealing approach for privacy-preserving analytics. However, its practicality is constrained by a limited set of supported operations—primarily additions and multiplications. This fundamental limitation necessitates the approximation of activation functions using polynomials, introducing a critical trade-off among approximation accuracy, computational efficiency, and noise management. Consequently, nearly all prior attempts at HE-based machine learning and deep learning analysis have incurred more or less accuracy loss. Some try to maximize efficiency but at the cost of a more significant loss in accuracy. For example, Podschwadt and Takabi [30] proposed a noninteractive RNN prediction protocol using parallel RNN blocks, which minimizes the number of communication rounds at the cost of a 5%-9% *F*_1_-value drop; Tosevski and Gulak [31] proposed an RNN quantization procedure to reduce the required HE computation, but their top-5 accuracy of secure speaker identification decreases from 0.9252 to 0.7805. While some other HE-based solutions achieved similar time-series model performance to nonsecure counterparts [32-34], it is crucial to note that a majority of these works focus solely on secure inference—performing predictions on an already-trained model. This approach presupposes the existence of a central, nonencrypted model, which is precisely the scenario that multiparty collaboration aims to avoid. The more challenging and impactful problem of secure training—learning a model from scratch on distributed, encrypted data without ever decrypting it—remains largely unaddressed for RNNs. A limited number of studies have ventured into secure HE-based RNN training, among which Paul et al [35] proposed a collective learning protocol on multiple locally trained RNN models. However, it is essentially a logistic regression training (only fully connected layer parameters change during the protocol) and requires direct sharing of local parameters, which may lead to privacy breaches. Another solution proposed by Sav et al [36] is more similar to ours. They extended ring learning with errors (RLWE)–based HE to the multiparty RNN training scenario by the use of threshold HE, where the decryption key is shared among participants. This introduces a critical new vulnerability: recent attacks have demonstrated that partial decryption shares can leak information about the secret key [37]. The primary countermeasure—injecting significant noise during the partial decryption phase—significantly erodes the precision of the underlying plaintext message, often rendering the model unusable, thus creating an unresolved tension between security and utility in multiparty HE-based training [38]. This unresolved tension between security and precision constitutes a major obstacle for practical multiparty HE-based training.

### Study Objectives

To overcome these limitations, this paper proposes a privacy-preserving medical AI analysis framework for distributed longitudinal patient data based on approximated threshold HE [39]. The training process is performed among multiple data providers and a researcher, where all shared intermediate results are encrypted or masked, and only the final model parameters are exposed to certain parties. To achieve practical efficiency without compromising security, we introduce several innovations: (1) a novel matrix multiplication scheme under HE for fewer multiplication depth possessions with very little waste of ciphertext slots; (2) a secure ciphertext refresh strategy that further reduces depth requirements, preserves ciphertext precision, and is proven secure under the indistinguishability under chosen plaintext attack with decryption oracles (IND-CPA^D^) security definition against a majority of dishonest passive adversaries [40,41]; and (3) the use of low-degree polynomial and piecewise functions to approximate activations effectively. Additionally, we use a sliced network structure to enhance convergence and accuracy for long sequences [42]. We implement and evaluate our framework on 4 real-world longitudinal medical datasets to assess its security, scalability, and predictive performance relative to nonsecure centralized training.

## Methods

### Ethical Considerations

This study involved secondary analysis of 4 publicly available, deidentified datasets: one from PhysioNet Challenge 2012, one from PhysioNet Challenge 2019, and two from MIMIC-III (Medical Information Mart for Intensive Care–III).

The MIMIC-III database was approved by the institutional review boards (IRBs) of the Beth Israel Deaconess Medical Center and Massachusetts Institute of Technology [43]. The PhysioNet Challenge 2012 dataset was collected from MIMIC-II, which was approved by the same IRBs of the MIMIC-III [44]. The PhysioNet Challenge 2019 dataset was derived from 3 hospital systems: Beth Israel Deaconess Medical Center, Emory University Hospital, and a third, unidentified one with approval from the appropriate IRBs [45].

This study used only publicly available, deidentified data and did not involve direct contact with participants. Therefore, no additional ethics approval or informed consent was required for this secondary analysis.

### Overview of the Proposed Protocol

**Figure 1.**
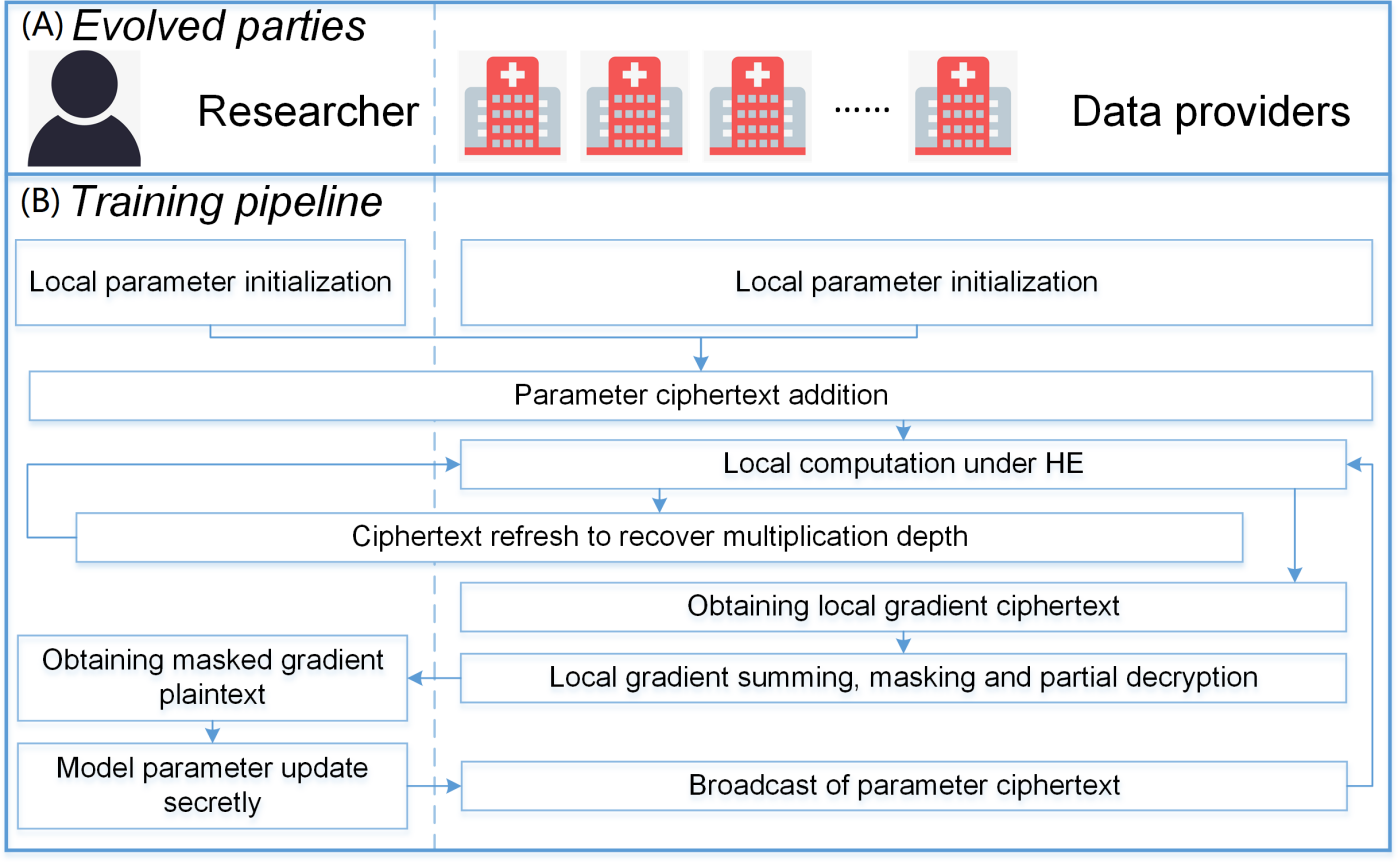
The architecture of the proposed privacy-preserving multiparty deep learning framework, where (A) shows evolved parties, including one researcher and multiple data providers, and (B) shows the pipeline of HE-based multiparty model training. HE: homomorphic encryption.

The architecture of our framework is shown in Figure 1, where two types of entities are mainly involved. One is data providers who hold longitudinal patient data and are willing to share it if no data leakage occurs. The other is a researcher who wishes to obtain a predictive model for various purposes like research or prediction while avoiding any exposure of model parameters to unauthorized parties. We assume that all medical data are transformed into a common format (eg, the OMOP [Observational Medical Outcomes Partnership] common data model [46]) to obtain correct multiparty research data.

As for adversary settings, given the number of involved parties *z*, up to *z* − 1 dishonest, passive adversaries can read the secrets of other corrupted parties while keeping the defined protocol running correctly. For the security of approximated HE, considering the revealing of partial decryption in threshold HE is similar to making a decryption query in normal single-key HE, we add Gaussian noises during ciphertext decryption and refresh according to Li’s work to defend IND-CPA^D^ attack [40], which was recently found to be a threat in both approximated and exact HE schemes, like Cheon-Kim-Kim-Song (CKKS) and Brakerski/Fan-Vercauteren (BFV) [47].

The pipeline of HE-based multiparty RNN model training includes the following steps. First, all parties initialize, encode, and encrypt their shares of model parameters, then secretly sum them into ciphertexts of initial model parameters, which will be broadcast to all data providers. Second, two steps are performed repeatedly until all encrypted local gradients are obtained: (1) each data provider conducts the forward and backward propagation process of the RNN model using its local data and ciphertexts of model parameters till the exhaustion of multiplication depth and (2) all parties together perform the online ciphertext refresh protocol to recover the multiplication depth. Third, all encrypted gradients are summed, masked, and partially decrypted (done by all data providers), and only the researcher gets the masked plaintext gradients. Lastly, the researcher secretly updates its model parameters without knowing the accurate gradients, which will be used in the next iteration. Furthermore, when the training process ends, the encrypted model parameters should be refreshed with a scale-up factor to counteract the impact of partial decryption noises on the accuracy of the decryption. We provide more details about ciphertext matrix multiplication, refresh, decryption, and sliced-RNN training below.

### Approximated HE and Optimization Techniques

HE is a type of encryption that allows computations on ciphertexts whose decrypted results can match the results of equivalent operations performed on plaintext. In our proposed framework, to perform secure model training, we use the RLWE-based HE scheme called CKKS that supports addition, multiplication, and vector rotation over the real number [48] (see more details in Multimedia Appendix 1 in the section “Threshold CKKS Scheme”). Since most of the operations over ciphertexts (eg, multiplication and vector rotation) heavily rely on polynomial multiplications, several optimization techniques are applied to maximize the efficiency of the CKKS scheme. These techniques include (1) fast polynomial multiplication based on the number theoretic transform [49], (2) the residue number system (RNS) representation that significantly reduces the computational cost of multiplication between two big integers [50], and (3) the batch encoding technique that makes a single ciphertext contain thousands of values, where any operation can be performed in a single instruction, multiple data (SIMD) manner [51]. Besides, we use a recent error reduction technique by Kim et al [52] so ciphertext rotation can be performed in smaller encryption parameters, and the threshold encryption technique is also integrated for multiparty applications [39]. Under threshold HE, each party holds a secret key, from which all collective keys in public are generated. When computation tasks start, all values will be encrypted by a collective public key, calculated by collective switching keys, partially decrypted by most of the parties, and finally decrypted by the remaining parties so that we can achieve access control of encrypted information and security against passive adversaries with a majority of dishonest parties.

**Figure 2.**
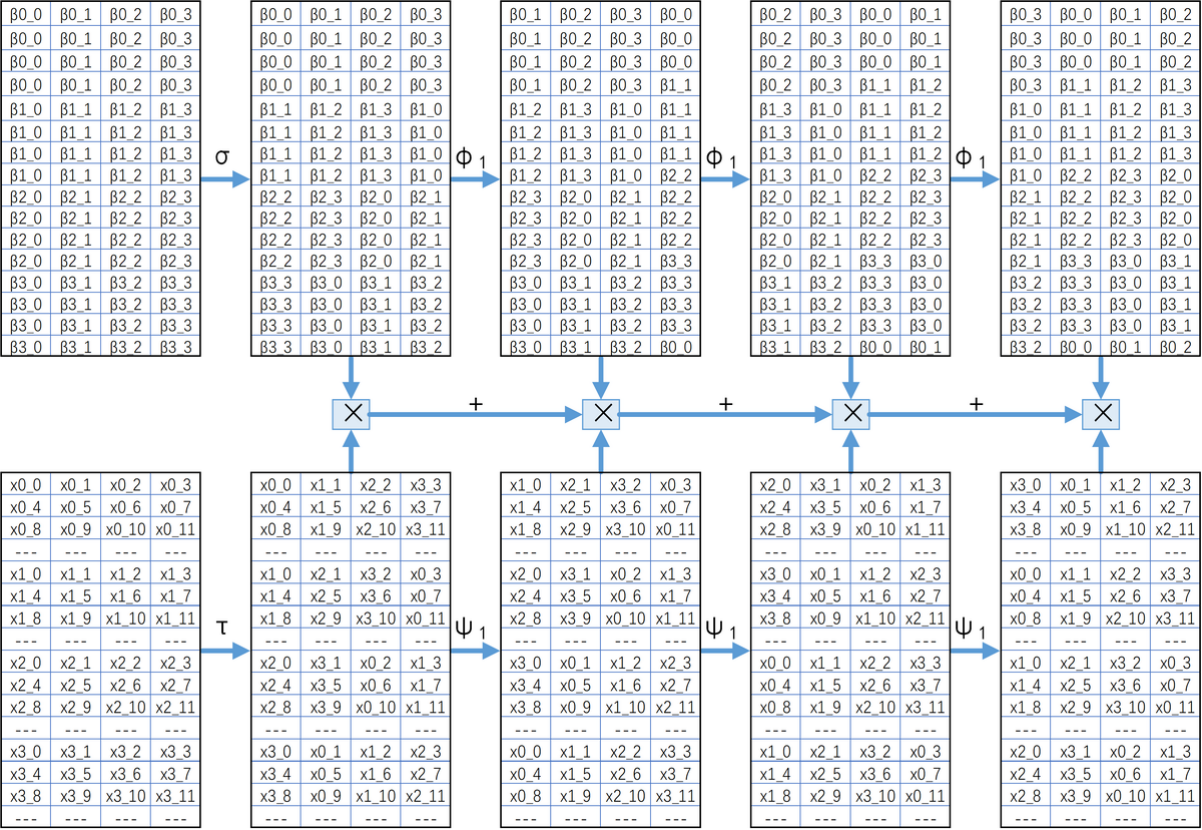
Schematic diagram of ciphertext matrix packing and multiplication for *l*=64 and *d*=4. Totally, two multiplication depths are required, one for the transformation operation *σ* and *τ* (while Φ_1_ and Ψ_1_ require only ciphertext rotations), the other for final *d*-times SIMD ciphertext multiplications. According to the values of *l* and *d*, the figure’s usage rate of ciphertext slots is 75%. SIMD: single instruction, multiple data.

Then, to optimize matrix multiplication over ciphertexts, we propose a novel matrix packing strategy that is suitable for batch and mini-batch gradient descent in common deep learning algorithms, as shown in Figure 2. Suppose an input matrix *x* and a model parameter matrix *β* whose sizes are *d*×*v* and *d*×*d*, respectively (the batch size is usually quite large to make full use of ciphertext slots, meaning that in many cases *v*>*d*), we copy the model parameters row by row and encode the input layer as follows. Let *m_i_* denote the *i*th element of an encoded ciphertext, *x_i_*___*_j_* denote the element of the input layer in the *i*th feature and *j*th sample, *l* denote the slot count of one ciphertext, and we assume that *l*/*d*^2^ is an integer, we have:


(1) $$\begin{array}{c} x_{i\_j}=m_{\frac{l\cdot i}{d}+j}\;\;\;\;\;i\in \left[0,d\right],j\in \left[0,\frac{l}{d}-\frac{l}{d^{2}}\right] \end{array}$$

Compared to the existing encrypted matrix multiplication protocol proposed by Jiang et al [53], our protocol has the following two advantages: (1) the number of ciphertext rotations is reduced from $3d+5\sqrt{d}$ to $2d+5\sqrt{d}$ with the baby-step giant-step algorithm; (2) the occupied multiplication depth is reduced from 3 to 2, which is much more important, meaning that we can perform more operations with the same encryption parameters or the same operations with smaller encryption parameters. The only drawback of our strategy is reducing the usage rate of ciphertext slots from 1 to $1-d^{2}/l$, which is acceptable since *d* is usually small and one ciphertext can contain at least thousands of values. By contrast, Rizomiliotis and Triakosia [54] recently proposed an encrypted matrix multiplication protocol with two multiplication depths but at the expense of a vast waste of ciphertext slots, which is not practical in data-intensive scenarios. Furthermore, when the size of the model parameter matrix becomes quite large, our packing strategy can be combined with the divide-and-conquer optimization scheme to minimize the impact of slot waste. Specifically, we can equally split the matrix of size *d*^2^ into much smaller matrices of size d'^2^ to increase the usage rate of ciphertext slots from $1-d^{2}/l\;to\;1-d^{\prime 2}/l$.

**Figure 3.**
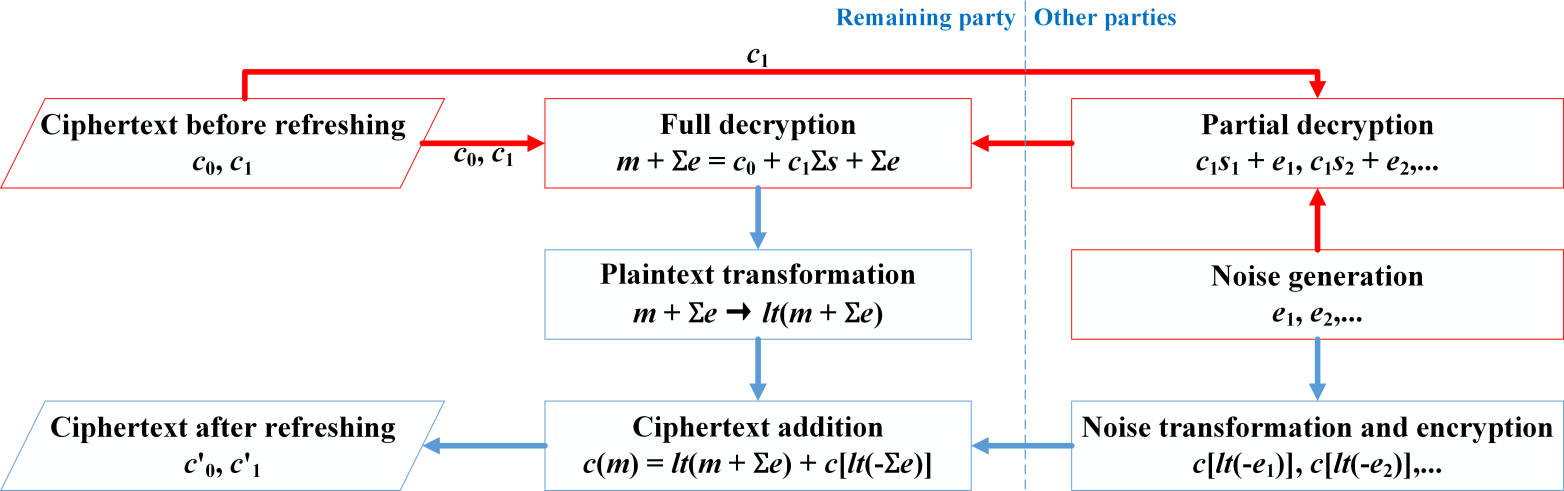
Pipeline of online ciphertext refresh (both the red and blue border/arrow) and decryption protocol (only the red border and arrow).

In the CKKS scheme, one ciphertext’s multiplication operation is limited. Once exhausted, the ciphertext refresh operation should be executed to recover the multiplication depth. Typically, it is done by a time-consuming technique called bootstrapping, whose efficiency is severely dragged down by many linear transformation operations, the fitting of the mod function using high-degree polynomials, and the need for large encryption parameters. Instead, considering the trade-off between computation and communication, we propose an online protocol to securely refresh ciphertexts without plaintext exposure, as shown in Figure 3. Initially, all parties but one generate partial decryption shares and add smudging noises. The noises should be sampled from a relatively large Gaussian distribution to avoid a full key recovery attack (which primarily exploits the approximate decryption results and is implied by IND-CPA^D^ security) and the leakage of plaintext simultaneously [40]. Next, all shares are sent to the remaining party. Then, according to the received shares, the remaining party fully decrypts the ciphertexts, obtains the masked plaintexts, and transforms the plaintexts as wished. Simultaneously, the same transformations are performed on all generated noises, which will be encrypted and sent to the remaining party later. Finally, after receiving the transformed encrypted noises, the remaining party performs addition among them and masked plaintexts to recover the original information secretly. By allowing transformation on plaintexts, we can perform matrix transpose and the first step of matrix multiplication out of the HE environment, thus eliminating many ciphertext rotations and further reducing the multiplication depth required for matrix multiplication from 2 to 1, which greatly reduces the noise generated during HE computations. Besides, an extra round of key generation should be performed after a certain number of ciphertext refreshes to maintain the IND-CPA^D^ security, which is not time-consuming if the encryption parameters are small.

Since the exact variance of the smudging noise is contingent upon the noise inherent in the decrypted ciphertexts, a preliminary local simulation is imperative. Prior to the multiparty RNN analysis, each data owner conducts a local training simulation on a small subset of their plaintext data. This simulation strictly adheres to the agreed-upon data preprocessing and model initialization protocols. Its objective is to determine the maximum output bounds for each layer, which in turn informs a static estimation of the noise growth per layer during subsequent homomorphic computations.

Like the online ciphertext refresh protocol, when the model training process ends, the final model parameter decryption also includes noise generation, partial decryption, and full decryption, with the researcher acting as the remaining party. Without adding transformed noises, we refresh model parameter ciphertexts with a large scale-up factor before decryption to avoid the impact of decryption noises on decryption accuracy.

### Privacy-Preserving RNN Model Training

Here, we describe how to perform RNN model training securely in two aspects: (1) the low-degree polynomial and piecewise fitting of activation functions and (2) the network structure used in conjunction with the fitting polynomials for better accuracy and convergence without too much computational overhead.

In nearly all HE-based machine learning and deep learning algorithms, a major challenge is the evaluation of nonpolynomial activation functions. All fitting strategies (eg, Taylor series, least squares error, and minimax technique) can only guarantee a reasonable approximation level within limited intervals. Once the input exceeds them, the error will grow dramatically, leading to the possible failure of model training, and things are usually even worse in RNN-based solutions due to the exploding or vanishing gradients. Therefore, selecting appropriate activation functions that are suitable for training tasks and easy to approximate is essential.

**Figure 4.**
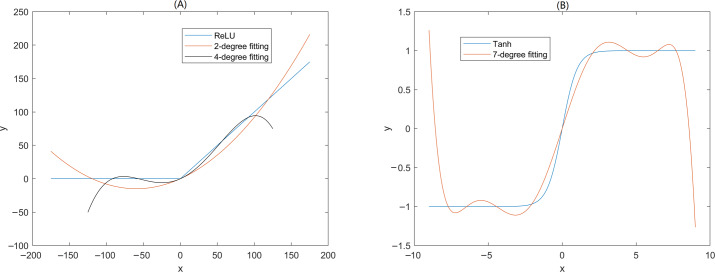
Comparison between approximated ReLU (A) and tanh (B). ReLU: rectified linear unit.

For the hidden layer in RNNs, tanh is a commonly used activation function, but its curve is quite steep if the input is close to 0 and very flat elsewhere. When fitting functions with such property, a polynomial with a higher degree should be applied to maintain a sufficient approximation interval at the cost of considerable computational overhead, or the accuracy loss of analysis results is likely to be significant, as demonstrated in previous work [30]. Thus, we choose the rectified linear unit (ReLU) function and use a quadratic function *f*_2_(*x*) and a 4-degree function *f*_4_(*x*) to approximate it with an intercept (0, 0) and least squares error, as shown in Figure 4.


(2) $$f_{2}(x)=0.5x+0.0042x^{2}$$

(3) $$\begin{array}{c} f_{4}\left(x\right)=0.5x+0.0109x^{2}-0.000000647x^{4} \end{array}$$

The occupied multiplication depths are only 1 and 2, and the approximation intervals are (−150, 150) and (−100, 100), which are obtained empirically and should be enough for model training on longitudinal patient data with most inputs within them. Even if a few inputs are outside the interval, it will not have a severe impact due to the slow relative error growth of the low-degree function compared to the fitting of sigmoid and tanh (usually at least 7 degrees), making the approximation more robust. Meanwhile, another restriction about the intercept is used to control the range of gradients in early iterations to avoid early gradient exploding.

However, in the output layer, the activation function mainly depends on the type of prediction tasks. For instance, if the task is binary classification, then the sigmoid function will usually be selected, and in our experiment, even a 15-degree least squares fitting polynomial may fail to converge. Therefore, we propose a secure piecewise linear approximation to maintain the boundedness of the original activation function without the leakage of actual predictive values, whose function and pipeline are shown below:


(4) $$y=\left\{ \begin{array}{ll} a_{1}x+b_{1} & x§lt;d_{1}\\ a_{i}x+b_{i} & x\in [d_{i-1}d_{i}],\;i\in [2,n-1]\\ a_{n}x+b_{n} & x\geq d_{n-1} \end{array}\right.$$

First, inner products between the input values of the output layer and the output layer’s model parameters are computed under HE to obtain the inputs of activation function *c*(*x*), where one value may appear in multiple ciphertext slots according to the number of linear regions. Next, these calculated inputs are subtracted with discontinuous points ${d=d}_{0},d_{1},\ldots ,d_{n-1}$ (*d*_0_=0, and the order is disturbed randomly) and multiplied by large uniformly distributed noises ${e=e}_{0},e_{1},\ldots ,e_{n-1}$ sampled from $U(-a_{i},a_{i})$ (*a_i_* is randomly selected from different distributions for each *e_i_*), which are stored locally and have the same order of discontinuous points. Then, the resulting ciphertexts are decrypted and refreshed, where the refreshed ciphertexts c(xe−de) and masked plaintexts xe-de are obtained by the researcher. After obtaining masked plaintexts, the researcher generates interval indicator plaintexts *ε* with only 0 and 1 (here, 1 and 0 indicate positive and negative values in masked plaintexts, respectively), encrypts them (denoted by c(ε(xe−de))), and sends these ciphertexts together with refreshed ones to corresponding data providers. Then, data providers also generate their binary indicator plaintexts ε(e) according to multiplicative noises and finally compute the piecewise linear approximation secretly as follows, which uses only two multiplication depths and can be easily implemented by “rotate and sum” operations:


(5) $$\begin{array}{l} c(y)=\frac{a_{1}}{e_{0}}c(xe_{0})+b_{1}+\sum_{i=1}^{n-1}{slope}_{i}\cdot (2\epsilon (e_{i})-1)\cdot \\ \;c\;\left(\epsilon (xe_{i}-d_{i}e_{i}\right)+\epsilon (e_{i})-1)\cdot c(xe_{i}-d_{i}e_{i}) \end{array}$$

(6) $$\begin{array}{c} slope_{i}=\frac{a_{i+1}-a_{i}}{e_{i}}+b_{i+1}-b_{i}+d_{i}\left(a_{i+1}-a_{i}\right) \end{array}$$

During the process, the only exposed information is masked inputs of the output layer’s activation function, from which an attacker is unlikely to infer any valid private data due to the zero-centered noises *e* and random order of e(x−d). The researcher can also benefit from them by judging whether the absolute values of masked inputs have grown too large, which usually means gradient explosion, thus stopping the failed training in the early stage and significantly avoiding the waste of computational resources. The main limitations of this HE-based piecewise linear approximation are (1) the need for an extra round of communication, which can coincide with ciphertext refresh by designing the forward propagation algorithm of secure sliced-RNN (see more details in Multimedia Appendix 1 in the section “Secure RNN Example”) and (2) the low utilization rate of ciphertext slots, which is inversely proportional to the number of linear regions, but in many real-world medical deep learning analysis scenarios, it is still acceptable since the number of outputs in the output layer is usually much smaller than that in the hidden layer.

As for network structure, we apply a sliced-RNN architecture when the time step is large, which splits the long input sequence into multiple equal-length subsequences and uses a traditional RNN-like structure on each subsequence. Then, all hidden states at the end of each subsequence are treated as a long sequence, which is again equally partitioned with the RNN-like structure. The split-forward cycle will repeat several times until the final sequence length reaches the requirement for model prediction [42]. Due to its parallel short subsequences and multiple hidden layers, it has some advantages that may be more prominent in the HE environment. (1) Compared to traditional RNNs, because one hidden layer’s all subsequences can be calculated simultaneously, sliced networks are more parallelized (especially in lower layers), meaning that they can make use of ciphertext slots more efficiently in most cases and are better adapted to various kinds of parallelization acceleration as well as the newly proposed ciphertext matrix multiplication protocol. (2) Sliced-RNN typically can obtain more information about long-term relationships through multiple hidden layers with little computational cost, which is very important in computationally expensive HE-based scenarios. However, more complex network structures and more model parameters can also lead to overfitting problems in noisy data. Meanwhile, low-degree polynomials typically provide limited ability to approximate nonlinear relationships compared to nonpolynomial activation functions like ReLU and tanh, possibly causing slower convergence and underfitting problems. Therefore, the combination of them may have a good trade-off.

## Results

### Experimental Setup

Here, we use three main aspects to evaluate the performance of our proposed multiparty longitudinal patient data modeling protocol: (1) *predictive accuracy*: comparing accuracy among secure, centralized nonsecure, and single-site nonsecure RNN model training, including original and sliced network structure; (2) *efficiency and scalability*: the time needed to perform secure sliced-RNN model training and its growth with data size and training settings; (3) *security*: whether sensitive data and trained models are at risk of exposure.

Four datasets are used for the experiments, which are described below. The first comes from PhysioNet Challenge 2012 [44], containing records of 12,000 intensive care unit (ICU) stays of adult patients. Given the test values within the first 48 hours, the target is to predict in-hospital mortality. The second comes from PhysioNet Challenge 2019 [45], where we randomly select 8000 ICU stays with at least 48 rows of measurements. For every ICU stay, we only use the last 48 rows, and the target is to predict the occurrence of sepsis. The third and last one is obtained from the MIMIC-III dataset, where the former contains test values from 18,415 patients during the first 8 days of hospitalization and aims to predict the 28-day mortality, and the latter includes 48 minutes long densely collected information about blood pressure, heart rate, and respiration from 5465 patients and is used for prediction of acute hypotension episodes [43]. Each time step is 1 hour in the first two datasets, 1 day in the third dataset, and 1 minute in the last one, making them have 48, 48, 8, and 48 time steps, respectively. Finally, data standardization is performed on all datasets to transform the range of different features into the same scale, and for missing values, the last observation carried forward is applied if a previously observed value exists. Otherwise, mean imputation is used.

For the encryption parameter setting of approximated HE, the selection is shown below to achieve 128-bits of IND-CPA^D^ security and (123, 32)-bits of 2^15^-IND-CPA^D^ security, which match the most recent work about IND-CPA^D^ and the security standards proposed by HomomorphicEncryption.org group [40,55]. The degree of polynomial modulus is 8192, while the coefficient modulus is a product of 7 distinct 31-bit primes. The multiplication depth of the HE scheme is 4, so we choose a 31-bit-long integer as the scale factor. The key, error, and decryption distributions follow *U*{–1, 0, 1}, *N*(0, 3.2^2^), and *N*(0, 2^64.58^), respectively.

For a real-world scenario, we implement our framework on different machines with Windows 10, each representing a data provider or researcher. The machines have AMD Ryzen 5 5600X CPUs running at 3.7 GHz, with 12 threads on 6 cores and 32 GB of RAM. Communication between any two machines is done through the Transmission Control Protocol with a bandwidth of 1 Gbps. To achieve the threshold variants of the CKKS scheme and support privacy-preserving multiparty sliced-RNN model training, we use the Microsoft Simple Encrypted Arithmetic Library v4.1.1 and have made some modifications [56].

### Accuracy Comparison

We first evaluate the impact of ciphertext noises on the predictive accuracy of the trained model, where the noises are produced during encryption, decryption, and computational operations. Here, we use the PhysioNet Challenge 2012 dataset and a stratified random sample of 2/3 for the training set and 1/3 for the testing set. Given the same initialized model parameters and sliced-RNN configuration (number of features *f*=39, number of hidden layer and output layer’s dimensions h,o=31, mini-batch size b=250, learning rate $\alpha =0.001\times 0.5^{0.01\times epoch}$, time step setting T=[48,2,2,2,2,3], where the first number indicates the total time steps and the following ones mean the minimum subsequence of each hidden layer, number of epochs ep=25), the noise estimation and area under the curve (AUC) comparison are shown in Table 1. Here, both the inherent noise of ciphertexts and smudging noise have nearly no impact on model performance. Therefore, to accelerate the accuracy evaluation of our framework, we perform it in a pseudo-HE manner, which replaces all activation functions with approximated polynomial (ReLU, 2-degree for the first three datasets and 4-degree for the remaining one) and piecewise linear (sigmoid) functions, and still calculates in plaintext but limits all computation operations to addition, multiplication, and vector rotations.

**Table 1.** Noise estimation and accuracy comparison between noise and noise-free solutions. The average difference in model parameters is 0.00029.

Solution	Noise estimation	Upper bound of hidden layer	Smudging noise	AUC^a^
Noise	22.48 bits	25 (35.64 bits)	54.77 bits	0.849157
Noise-free	—^b^	—	—	0.849154

a AUC: area under the curve.

b Not applicable.

Then, to demonstrate the model prediction performance of our proposed framework, we conduct a comparative experiment using all above-mentioned longitudinal patient datasets with (1) nonsecure RNN with ReLU or tanh in the hidden layer and all data centralized in 1 machine, where ReLU is closer in shape to our low-degree polynomials, and tanh is more commonly used in RNN, (2) nonsecure sliced-RNN with ReLU in hidden layers and all data centralized in 1 machine, and (3) secure RNN with data distributed in multiple machines with replaced polynomial activation functions like ours. The training settings are shown in Table 2, which repeats 50 times using 10 different random seeds for the stratified sampling of the training and testing set, and for each stratified sampling, 5 different random seeds are used for model parameter initialization (all use the same random seeds in one experiment). Here, to achieve faster convergence and avoid the gradient explosion problem as much as possible, we choose Xavier initialization for parameters of the hidden layer in RNN (typically, He initialization should be used on the ReLU function, but in our experiment, it is significantly slower to converge and more vulnerable to gradient explosion in RNN) and output layer, and He initialization for parameters of the hidden layer in sliced-RNN. Class weights are also used to address the label imbalance of all datasets. Finally, after each epoch, the overall loss of the testing set is calculated secretly and decrypted for possible early stops, which usually obtain AUCs slightly lower than the theoretical maximum AUC.

**Table 2.** Training setting of 4 datasets in accuracy comparison.

Dataset	PhysioNet Challenge 2012	PhysioNet Challenge 2019	MIMIC^a^-III (28 days mortality)	MIMIC-III (acute hypotension episodes)
# of training set (# of positive one)	8000 (1138)	4000 (508)	12,000 (1409)	3000 (1292)
# of testing set (# of positive one)	4000 (569)	4000 (508)	6415 (753)	2465 (1061)
*f*	39	36	50	9
*h*, *o*	31	31	31	7
*b*	250	250	250	250
*α*	0.001 × 0.5^epoch/100^	0.0005 × 0.5^epoch/100^	0.001 × 0.5^epoch/100^	0.001 × 0.5^epoch/200^
*T*	[48,2,2,2,2,3]	[48,2,2,2,2,3]	[8,2,2,2]	[48,2,2,2,2,3]
Maximum epoch time	200	200	200	400

a MIMIC: Medical Information Mart for Intensive Care.

**Figure 5.**
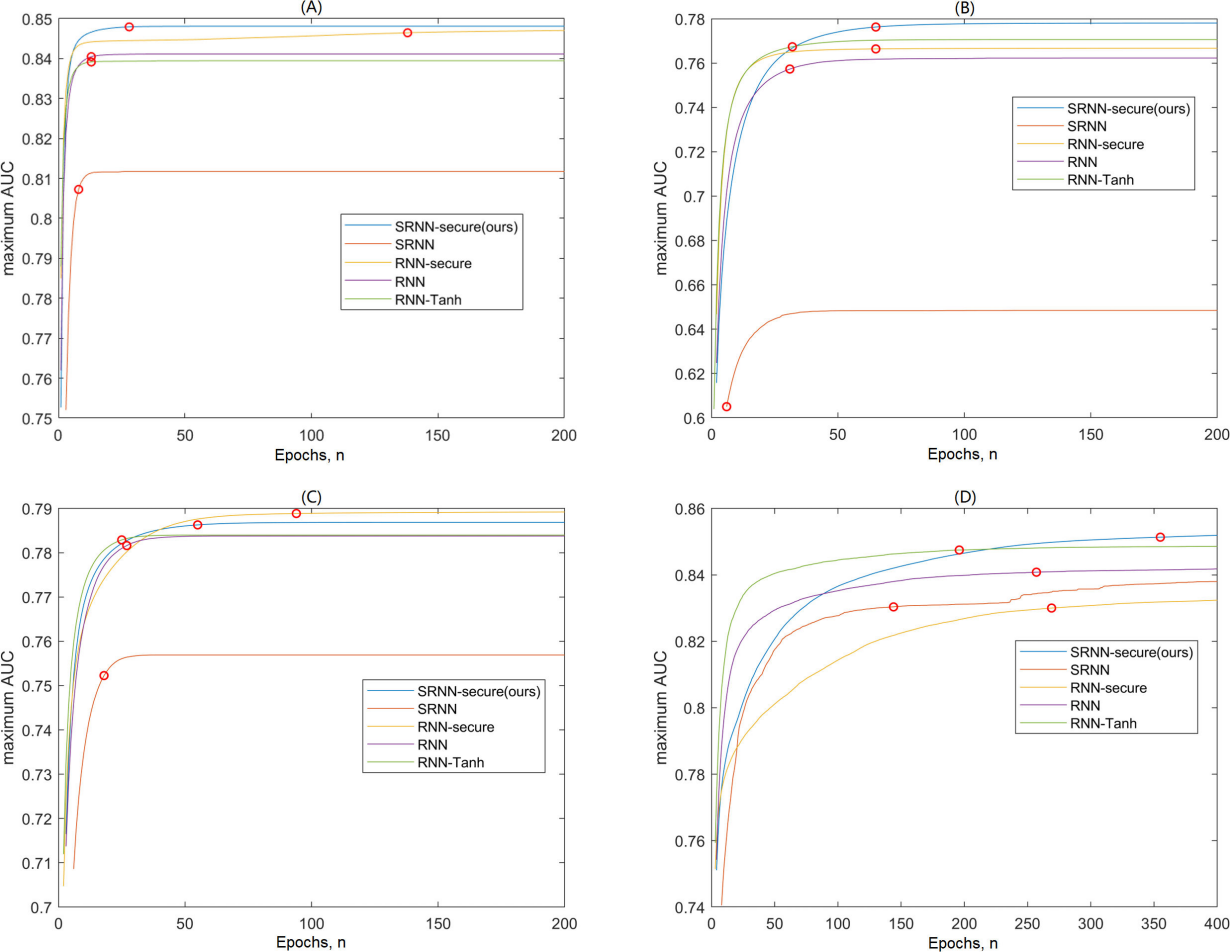
Comparison of testing sets’ maximum AUC variation with the number of epochs in (A) the PhysioNet Challenge 2012 dataset, (B) the PhysioNet Challenge 2019 dataset, (C) data extracted from MIMIC-III for 28-day mortality prediction, and (D) data extracted from MIMIC-III for acute hypotension episodes prediction. The red dots indicate the points at which each solution achieves a minimum overall loss of the testing set. AUC: area under the curve; MIMIC: Medical Information Mart for Intensive Care; RNN: recurrent neural network; SRNN: sliced recurrent neural network.

The experimental results are shown in Figure 5 and Table 3, where the former shows the variation of maximum AUC with the number of epochs for the proposed and compared solutions, and the latter lists their prediction performance and the corresponding number of epochs required to reach the minimum overall loss in the testing set (averaged over 50 repetitions of the experiments with different random seeds). In general, the use of low-degree approximated functions does lead to more epochs for near-optimal prediction performance, but surprisingly, the model predictive ability of secure solutions is overall a little better than nonsecure solutions, where the performance is relatively much closer in between nonsecure and secure RNNs and significantly different between nonsecure sliced-RNN and ours. The uncommonly low maximum AUC of the nonsecure sliced-RNN model may be related to the fact that its model structure is overly complex compared to traditional RNNs, while longitudinal clinical data tend to have much more missing and biased data than many other fields due to the unsuccessful measurements and specificity of patients during long observation windows (especially in the first 3 datasets since the interval between time steps is much longer than that in the last dataset) [57], which together lead to overlearning of the training set’s noisy features and severe overfitting. By contrast, our solution significantly mitigates the overlearning problem of the training set by limiting the ability to represent nonlinear relationships through low-degree polynomial activation functions, making it more robust against noisy features, thus obtaining even higher prediction performance on all 4 datasets compared to the original nonsecure RNN. It is also the first case, to the best of our knowledge, where the HE-based trained model has better predictive ability than the corresponding nonsecure one with the same training data, while similar previous studies have shown either significant or minor losses in accuracy [30,36].

Compared to the secure RNN, our solution also provides better training results overall, significantly outperforming it in the 1st, 2nd, and 4th datasets, either requiring only much fewer epochs while having similar prediction performance (1st dataset) or having explicitly better prediction performance with relatively closer epochs (2nd and 4th datasets). Notably, the 4th dataset has much shorter testing time intervals and fewer missing values than the other 3 datasets, in which secure RNN obtains the worst prediction performance, showing its limited ability to handle nonlinear relationships with less noisy features. In contrast, ours achieves a better trade-off and obtains the best prediction performance. On the other hand, the secure RNN’s trained model performs slightly better in the third dataset, which may be caused by the dataset’s much fewer time steps. Such short sequences are insufficient to fully utilize the long-term relationship acquisition advantage of sliced-RNN, while the sliced structure impairs neighboring relationships to some extent between parts of time steps.

Furthermore, to demonstrate the advantage of cross-institutional collaborative analysis over single-site analysis, we conduct a comparative performance evaluation between our secure sliced-RNN models (trained on the entire dataset) and nonsecure RNN models (trained on only a partial dataset) with the same test sets in Table 3. We simulated a realistic scenario where data are equally partitioned between two institutions (A and B) but with an imbalanced label distribution (positive rate ratio of 2:3). As shown in Table 4, our secure sliced-RNN model, trained on the entire dataset, demonstrates significantly superior predictive performance across all 4 medical datasets compared to models trained on isolated institutional data. Critically, this advantage is consistently observed under both label imbalance and balanced conditions. In the first 3 datasets, where positive cases represent only 10%-20% of the population, our model’s performance gain is occurred most in the AUC and recall—precisely the metrics most crucial for identifying rare but clinically critical events. For instance, on the PhysioNet Challenge 2012 dataset, SRNN (sliced recurrent neural network)-secure achieves a recall of 0.7981, dramatically outperforming both RNN A (0.5937) and RNN B (0.6057), demonstrating a markedly enhanced ability to identify true positive cases without being hindered by the class imbalance. This superior performance extends to the fourth dataset, which exhibits a balanced label condition. Here, SRNN-secure not only achieved the highest AUC but also excelled across all metrics including accuracy, recall, and *F*_1_-score, showcasing its robustness and ability to integrate diverse data patterns effectively.

**Table 3.** Training results among (1) traditional and sliced network structures and (2) nonsecure and secure solutions^a^.

Solution	Max AUC^b^, mean (SD)	Accuracy, mean (SD)	Recall, mean (SD)	*F*_1_-score, mean (SD)	Epochs, n
PhysioNet Challenge 2012					
SRNN^c^-secure	*0.8480 (0.0061)*	0.7575 (0.0240)	*0.7981 (0.0061)*	0.4846 (0.0163)	27.78
SRNN-nonsecure	0.8065 (0.0143)*^d^	0.7272 (0.0267)*	0.7600 (0.0391)*	0.4430 (0.0182)*	7.76
RNN^e^-secure	0.8468 (0.0073)	*0.7655 (0.0213)*	0.7869 (0.0318)*	*0.4892 (0.0148)*	137.64
RNN-nonsecure	0.8404 (0.0076)*	0.7608 (0.0235)	0.7805 (0.0386)*	0.4823 (0.0146)	12.98
RNN-tanh	0.8393 (0.0076)*	0.7616 (0.0198)	0.7795 (0.0277)*	0.4827 (0.0151)	12.56
PhysioNet Challenge 2019					
SRNN-secure	*0.7762 (0.0118)*	*0.7540 (0.0285)*	0.6715 (0.0432)	*0.4107 (0.0176)*	65.04
SRNN-nonsecure	0.6030 (0.0378)*	0.6726 (0.0721)*	0.4716 (0.1131)*	0.2671 (0.0323)*	5.64
RNN-secure	0.7661 (0.0111)*	0.7328 (0.0276)*	0.6765 (0.0352)	0.3924 (0.0161)*	64.50
RNN-nonsecure	0.7576 (0.0137)*	0.7263 (0.0311)*	0.6669 (0.0455)	0.3834 (0.0172)*	31.20
RNN-tanh	0.7669 (0.0125)*	0.7204 (0.0300)*	*0.6869 (0.0385)*	0.3854 (0.0170)*	31.68
MIMIC^f^-III dataset for 28-day mortality prediction					
SRNN-secure	0.7865 (0.0073)	0.7214 (0.0238)	0.7187 (0.0313)	0.3779 (0.0125)	55.46
SRNN-nonsecure	0.7516 (0.0157)*	0.6855 (0.0378)*	0.6976 (0.0492)*	0.3437 (0.0160)*	17.50
RNN-secure	*0.7890 (0.0077)*	*0.7223 (0.0300)*	0.7221 (0.0404)	*0.3802 (0.0146)*	94.28
RNN-nonsecure	0.7821 (0.0092)*	0.7070 (0.0315)*	0.7203 (0.0453)	0.3669 (0.0129)*	27.44
RNN-tanh	0.7833 (0.0090)*	0.7034 (0.0260)*	*0.7303 (0.0349)*	0.3670 (0.0121)*	25.46
MIMIC-III dataset for acute hypotension episodes prediction					
SRNN-secure	*0.8518 (0.0074)*	*0.7941 (0.0096)*	0.8209 (0.0252)	*0.7744 (0.0088)*	354.88
SRNN-nonsecure	0.8356 (0.0185)*	0.7829 (0.0150)*	*0.8345 (0.0332)*	0.7680 (0.0108)*	143.94
RNN-secure	0.8321 (0.0203)*	0.7750 (0.0186)*	0.8261 (0.0319)	0.7598 (0.0138)*	269.30
RNN-nonsecure	0.8415 (0.0127)*	0.7867 (0.0120)*	0.8232 (0.0286)	0.7687 (0.0094)*	256.50
RNN-tanh	0.8479 (0.0103)*	0.7914 (0.0119)	0.8240 (0.0265)	0.7728 (0.0100)	195.66

a Values in italic indicate the highest mean.

b AUC: area under the curve.

c SRNN: sliced recurrent neural network.

d * indicates the values are significantly smaller (*P*<.05) than that of our solution (SRNN-secure).

e RNN: recurrent neural network.

f MIMIC: Medical Information Mart for Intensive Care.

**Table 4.** Training results between (1) secure sliced-RNN models on the entire dataset and (2) nonsecure RNN models on the partial dataset^a^.

Solution	Max AUC^b^, mean (SD)	Accuracy, mean (SD)	Recall, mean (SD)	*F*_1_-score
PhysioNet Challenge 2012				
SRNN^c^-secure	*0.8480 (0.0061)*	0.7575 (0.0240)	*0.7981 (0.0061)*	0.4846 (0.0163)
RNN^d^ A	0.8245 (0.0091)*^e^	0.8278 (0.0143)	0.5937 (0.0432)*	0.4953 (0.0124)
RNN B	0.8295 (0.0096)*	*0.8280 (0.0158)*	0.6057 (0.0428)*	*0.5008 (0.0119)*
PhysioNet Challenge 2019				
SRNN-secure	*0.7762 (0.0118)*	0.7540 (0.0285)	*0.6715 (0.0432)*	*0.4107 (0.0176)*
RNN A	0.7242 (0.0196)*	0.7950 (0.0301)	0.4767 (0.0631)*	0.3719 (0.0221)*
RNN B	0.7427 (0.0125)*	*0.8017 (0.0222)*	0.4994 (0.0520)*	0.3904 (0.0157)*
MIMIC^f^-III dataset for 28-day mortality prediction				
SRNN-secure	*0.7865 (0.0073)*	0.7214 (0.0238)	*0.7187 (0.0313)*	0.3779 (0.0125)
RNN A	0.7461 (0.0167)*	*0.8170 (0.0241)*	0.4610 (0.0530)*	0.3721 (0.0126)*
RNN B	0.7612 (0.0138)*	0.8105 (0.0255)	0.4903 (0.0554)*	*0.3786 (0.0139)*
MIMIC-III dataset for acute hypotension episodes prediction				
SRNN-secure	*0.8518 (0.0074)*	*0.7941 (0.0096)*	*0.8209 (0.0252)*	*0.7744 (0.0088)*
RNN A	0.7572 (0.0177)*	0.6912 (0.0260)*	0.7539 (0.0500)*	0.6775 (0.0217)*
RNN B	0.7658 (0.0164)*	0.7035 (0.0187)*	0.7662 (0.0534)*	0.6895 (0.0194)*

a Values in italic indicate the highest mean.

b AUC: area under the curve.

c SRNN: sliced recurrent neural network.

d RNN: recurrent neural network.

e * indicates the values are significantly smaller (*P*<.05) than that of our solution (SRNN-secure).

f MIMIC: Medical Information Mart for Intensive Care.

Notably, these results are achieved under a constrained simulation with only two institutions. We anticipate that this performance gap would be substantially wider in real-world multi-institutional collaborations involving more data providers with greater heterogeneity. Our framework effectively harnesses such diversity to build more robust and generalizable models, which is unattainable by any single institution working in isolation.

### Analysis of Long-Term Training Stability

The use of low-degree polynomial approximations (eg, quadratic functions for ReLU) raises concerns about gradient explosion over extended time steps, as the hidden input may exceed the fitting interval during the long-term forward and backward propagation, making the approximations completely unreliable. Therefore, to empirically validate stability, we conducted a stress test on the PhysioNet Challenge 2012 dataset by artificially extending the sequence length from 48 to 96 and 192 steps via linear interpolation of the original 48-step trajectories. Across 50 independent runs for each sequence length (the same random settings of Table 2, shown in Multimedia Appendix 1 in the section “Long-Term Training Stability With Approximated Activations”), we find that (1) no hidden input exceeds the fitting interval, even at 192 steps; (2) both the hidden input range and the $L_{\infty }$-norm of gradients show no significant increasing trend as sequence length grows (48-step: hidden input range [−41.560, 39.474], $L_{\infty }$-norm 0.0147; 192-step: hidden input range [−43.692, 43.297], $L_{\infty }$-norm 0.0142). These results confirm that our quadratic ReLU approximation provides stable training for sequence lengths up to at least 4 times the original 48-step limit. We attribute this stability to (1) the sliced-RNN architecture, which effectively limits the propagation depth; (2) the bounded nature of the input features after standardization; and (3) the smoothing effect of low-degree polynomial approximation, which reduces sensitivity to noise and mitigates overfitting.

### Time Consumption and Scalability

**Figure 6.**
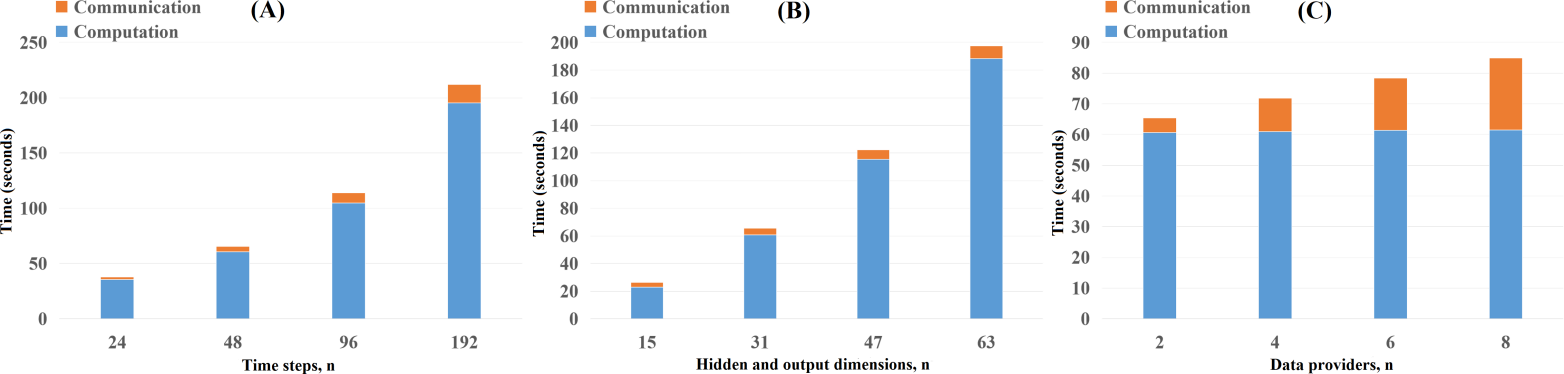
Scaling of time consumption in 1 iteration with (A) the number of time steps, (B) the number of hidden layer and output layer’s dimensions, and (C) the number of data providers (here we assume that each data provider maintains the same local mini-batch size, meaning the overall mini-batch size also grows proportionally). Default setting: 2 data providers, *f*=39, *h* and *o*=31, *b*=125×2, 48 time steps.

The scalability of our protocol is shown in Figure 6, where we test our framework in different experimental settings. Specifically, as the number of time steps grows, both the computation and communication time increase proportionally; as *h* and *o* grow, the computation and communication time increase near quadratically and linearly, respectively, since matrix multiplication and outer product computation form the majority of computational overhead and most weight matrices enlarge quadratically with hidden and output dimensions; in contrast, as the number of data providers grows (each of them maintains the same local mini-batch size), the communication time increases linearly, but each data provider’s computation time varies very little. These results are consistent with previous similar HE-based works [12,36], meaning that adopting the sliced structure does not negatively impact the scalability of time-series data model training. Besides, the lightweight encryption parameters and low multiplication depth of the fitting polynomials and matrix multiplication in our framework also effectively reduce computational overhead, with about 1 minute to perform one iteration of model training in real-world longitudinal patient data. In comparison, in Sav’s work [36], 7-degree polynomial fitting tanh and ciphertext matrix multiplication with 3 multiplication depths are used, which leads to much larger encryption parameters and operation times of ciphertext multiplication, thus requiring nearly the same time to perform one iteration of RNN model training in only much smaller settings in their experimental results (the same hidden dimensions, doubled local mini-batch size, and 4 time steps).

Furthermore, we conducted a simulation-based study to emulate various wide area network (WAN) conditions and estimate the impact of communication latency in our framework. Specifically, we artificially imposed three levels of round-trip time (RTT: 20, 50, and 100 ms) and evaluated the additional training time under different numbers of time steps and data providers for each latency setting. The results are presented in Multimedia Appendix 1 in the section “Impact on Communication Latency.” Notably, the additional training time increases linearly with the latency, and the growth is much less significant with the increasing of time steps and data providers. This is because the number of ciphertext communication rounds scales only logarithmically with the number of time steps due to the sliced network structure, and remains constant with the number of data providers, as the ciphertext refresh and partial decryption protocol requires a fixed number of communication rounds independent of party count. These findings demonstrate that our framework maintains good scalability under real-world WAN conditions and remains practical even under relatively high latency (eg, 100 ms), as the additional training time constitutes only a small fraction (approximately 10% in the default setting) of the total training time under nearly zero-latency conditions.

### Security Analysis

The security of our proposed framework is discussed in the following 4 aspects.

#### Secret Keys Held by Each Party

We consider two situations for using secret keys. One is collective key generation, and the other is partial and final decryption, where the security of secret keys in the former case has already been proved by the simulation paradigm with all but one colluding party [58]. In the latter case, the risk of secret keys arises mainly from (1) the possible exposure of secret keys due to the delivery of partial decryption shares and (2) a full key recovery attack specialized for approximate RLWE-based HE that is mainly initiated by the final decrypting party [40], both of which can be solved by adding large smudging noises to achieve a sufficient level of statistical security. In addition, when the number of ciphertext refreshes and decryption reaches a certain value, all keys will be regenerated to limit the decryption query times under every set of keys, thus ensuring IND-CPA^D^.

The noise estimation is non-worst-case, while Guo’s [37] attack showed that, only with worst-case noise estimation, the noise-flooding countermeasures can achieve IND-CPA^D^. However, in their adversary setting, the attacker can select arbitrary inputs and HE computation protocols, which is not allowed in passive adversaries [59,60]. Therefore, this attack is not applicable to our framework, and non-worst-case noise estimation is sufficient to overwhelm ciphertext noise and plaintext information (54.77 bits vs 22.48 and 35.64 bits, shown in Table 1).

#### Whether the Researcher Is Honest (Security of Model)

All data providers can only obtain masked intermediate results during the whole model training process, where the researcher partially generates the smudging noises. As long as the researcher’s noise shares are large enough (the size of noise added by each party during the ciphertext refresh process has nearly no impact on the accuracy of subsequent computation, so it is easy to achieve), an attacker will not be able to infer any valid training model information from them. Meanwhile, for initial model parameters, the initialization is performed by all parties, meaning that they cannot be directly exposed even with all corrupted data providers.

#### Whether a Data Provider Is Honest (Security of Its Data)

For an honest data provider, the only exposed information is noisy logit values, which are eventually decrypted by the researcher and used to determine the belonging intervals for piecewise linear fitting functions secretly. Since the zero-centered multiplicative noises and the order of discontinuous points are randomly generated only by the honest data provider, an attacker is unlikely to extract the exact logit values. Besides, the honest data provider can disrupt the order of data training for each epoch, making it very hard for an attacker to match multiple logit values of the same sample over multiple epochs, thus further avoiding the implied data leakage from such aggregated information.

#### Extension to Active Adversaries

While our current framework assumes passive adversaries, its design is compatible with extensions to handle active adversaries—who may deviate from the protocol by submitting manipulated gradients or false partial decryption shares. Recent advances have demonstrated the feasibility of combining fully HE with Byzantine-robust aggregation to defend against such malicious behavior. For example, Lancelot introduces a mask-based encrypted sorting mechanism that enables robust aggregation rules such as Krum and median to operate directly on encrypted model updates, effectively protecting against malicious clients that attempt to poison the global model [61]. It achieves this enhanced security with little computational overhead, taking only 18× the time of plaintext operations (compared to 384× for the open-source fully homomorphic encryption [OpenFHE] approach). Therefore, it can be integrated into our framework to provide cryptographic privacy and Byzantine resilience simultaneously without incurring prohibitive computational or communication overhead.

## Discussion

### Principal Findings

The above experimental results demonstrate that our proposed framework successfully achieves a practical trade-off among security, accuracy, and efficiency. First, it enables secure multiparty RNN training on distributed longitudinal patient data without exposing raw data or model parameters. Second, it maintains good stability in long-term datasets, shows significant accuracy improvement compared to simulated single-site analysis, and achieves predictive performance comparable to—in some cases even exceeding—nonsecure centralized models (eg, AUC of 0.8480 vs 0.8404 on the PhysioNet Challenge 2012 dataset). Third, it maintains practical efficiency, requiring approximately one minute per training iteration under realistic settings, with favorable scalability as data size and number of participants increase. These findings confirm the feasibility of privacy-preserving deep learning on medical time-series data using threshold HE.

### Interpretation, Implications, and Comparison With Prior Work

The integration of threshold approximated HE with noise engineering shows excellent potential. The large noises added by various parties during ciphertext refresh and decryption, together with the underlying plaintext message, effectively form a linear secret sharing scheme such as Mouchet’s collective bootstrapping protocol in the threshold BFV scheme, ensuring the security of the exposed masked values [58,62]. More importantly, we leverage this secret-shared plaintext domain to perform specific linear operations—namely, data scaling and encoding changes—that would otherwise require expensive homomorphic operations (specifically, ciphertext-plaintext multiplication and ciphertext rotation). This strategic optimization further enhances the multiplicative depth availability and overall ciphertext computation efficiency. Consequently, under a computable ciphertext environment, substantial noise can be injected into intermediate and final results during refresh and decryption without compromising computational precision. This approach guarantees the complete nonrecoverability of any exposed information and provides robust defense against statistical attacks targeting both threshold and approximated HE. In contrast, when relying solely on differential privacy, a challenging trade-off between security and accuracy invariably persists, particularly within RNN-like structures [26].

Our comparison of predictive ability between secure and nonsecure solutions also shows the promise of the proposed framework for high-quality multiparty privacy-preserving analysis on datasets with noisy and large parts of missing features, which is common in the biomedical field. Almost all previous HE-based machine learning and deep learning model training works have focused on fitting polynomials that are as close as possible to the original activation functions, mainly validated on high-integrity publicly available datasets, and expected to obtain analytical results that are either nearly the same as the corresponding nonsecure central solution or having an acceptable loss of accuracy with the same or a little larger number of iterations [12,30,36]. In contrast, we use more complicated networks and low-degree functions to fit the ReLU function with a large fitting interval, introducing a larger fitting noise, but achieving higher AUCs instead. This finding indicates that future HE-based iterative computing research may not only be simple mimics of existing algorithms but rather a comprehensive optimization problem combining data, polynomial activation function selections, network structure, and neuron structure, which can be very challenging and needs further exploration.

The significant difference between initial settings of maximum epoch times and final experimental obtained early stop points also indicates the importance of exposing specific information in HE-based iterative computing solutions due to the time-consuming and invisible nature of the calculation. For example, after a certain number of epochs, a privacy-preserving inference should be made on the testing set (well suited to our proposed matrix multiplication, as the number of testing samples is usually much larger than the batch size), and then the plaintext of some testing set’s aggregated information should be obtained by the researcher to facilitate timely stop of training, rather than empirically specifying hyperparameters, and suffering useless model training, or even overfitted results, leading to waste of a large amount of computational, storage, and network resources. Here, for a better trade-off among efficiency, security, and utility, we select overall loss as the indicator since it is straightforward to compute under an HE environment, very close to the theoretical best prediction performance (at least in our secure sliced-RNN, as shown in Figure 5), and reveals only one value per computation, which is hard for attackers to infer private information. On the contrary, if we directly use prediction performance (like AUC) as an indicator, the computation in the HE environment will be much more complex and impractical because of the limitations of ciphertext operations and efficiency.

Another advantage of our framework is the potential to adapt well to longitudinal patient data with different overfitting risks and task types without additional overfitting prevention settings. In comparison, HE-based solutions with perfectly fitted activation functions (as nonsecure ones, for example, using a proposed piecewise linear function to perform ReLU) are more or less affected by overfitting and can only obtain comparable prediction performance to ours when using overfitting prevention measures (such as L2 regularization) with proper hyperparameters at the cost of obviously more epoch times overall (see more details in Multimedia Appendix 1 in the section “Performance With L2 Regularization”). This is essential in privacy-preserving real-world medical data analysis scenarios, where researchers often do not have direct access to research data and, therefore, have difficulty determining overfitting prevention hyperparameters. Federated tuning methods have problems with privacy and heterogeneity: private information leakage mainly comes from sharing of private local model parameters, weights, and optimal hyperparameters [20,21,63], while heterogeneity makes it difficult to approach the real global optimum and is quite common in the medical research domain since different medical institutions often have different treatment abilities, scopes of patients, and data collection strategies, causing distinct feature, label, quantity, and noise distributions among all data providers [64,65]. Centralized tuning methods like grid search and Bayesian optimization are accurate, but the operation time may grow very large with extra hyperparameters due to their iterative nature and the expensive computational cost of HE operations. Therefore, with fewer hyperparameters, our solution may save substantial time when used in the real world.

### Limitations

Here, we test our framework in many-to-one scenarios, but in the real world there are quite a few many-to-many scenarios as well (eg, prediction of acute exacerbation at fixed time intervals during hospitalization), where if we continue using the proposed secure piecewise linear approximation, an excessive number of ciphertexts may be produced to lower the computational efficiency due to the low utilization of ciphertext slots. In this case, some alternative polynomial fitting techniques exist for large intervals. For instance, the domain extension algorithm by Cheon et al [66] can extend the valid fitting interval of an approximated polynomial by a factor of 2.45 for every two multiplicative depths consumed, which is very useful when the original polynomial is high-degree (eg, for sigmoid, 9-degree or higher), thus requiring larger encryption parameters or more rounds of ciphertext refresh. As for ciphertext matrix multiplication, Jang et al [33] recently proposed a variant of the CKKS scheme called MatHEAAN, which directly supports ciphertext rotations in a matrix manner and occupies only two multiplication depths without losing any ciphertext slots. However, as it is not yet integrated with the RNS, its current efficiency does not surpass our RNS-based scheme, though future advancements may make it a viable alternative. Besides, while our stability analysis demonstrates robustness up to 192 steps, we acknowledge that the interpolated sequences may not fully capture the statistical properties of real clinical time series of such lengths, which will be further evaluated in the future.

### Conclusions

This paper proposes a protocol for privacy-preserving multiparty RNN analysis of longitudinal patient data using threshold HE. The experimental results demonstrate that secure training can achieve even higher predictive accuracy compared to nonsecure centralized models in some cases, with practical efficiency and good scalability. More broadly, these findings challenge the prevailing assumption that cryptographic privacy inevitably sacrifices accuracy, and instead reveal an unexpected synergy: low-degree polynomial approximations, chosen primarily for computational efficiency, also act as an implicit regularizer that mitigates overfitting on noisy clinical data. This insight points toward a new design paradigm for privacy-preserving medical AI—one where cryptographic protocols and model architectures are co-designed rather than naively combined. We believe that with existing clinical information integration platforms, our framework will promote multiparty longitudinal medical research and help accelerate knowledge discoveries.

Some future works still exist to make it more practical. First, GPU (graphics processing unit) acceleration can significantly improve the efficiency of basic ciphertext operations, while the software pipelining technique can better accelerate our framework than traditional RNNs since different subsequences can be executed in parallel under a sliced structure. Second, consider the use of more complex neuron structures and apply them to research scenarios where more data providers provide the longitudinal patient data, then explore the predictive performance of ciphertext computation solutions in different configurations on data with a varying degree of missing and noisy features. Furthermore, it is also essential to evaluate the impact of hyperparameter tuning with different heterogeneity in real-world medical data distribution scenes. If needed, an efficient privacy-preserving tuning method may be further researched.

## Supplementary material

10.2196/87306 **Multimedia Appendix 1.** Technical details of the threshold CKKS encryption scheme, secure sliced-RNN forward propagation algorithm, and supplementary experimental results. CKKS: Cheon-Kim-Kim-Song; RNN: recurrent neural network.

## Abbreviations

AUC: area under the curve
BFV: Brakerski/Fan-Vercauteren
CKKS: Cheon-Kim-Kim-Song
GDPR: General Data Protection Regulation
GPU: graphics processing unit
HE: homomorphic encryption
HIPAA: Health Insurance Portability and Accountability Act
ICU: intensive care unit
IND-CPA^D^: indistinguishability under chosen plaintext attack with decryption oracles
IRB: institutional review board
MIMIC: Medical Information Mart for Intensive Care
OMOP: Observational Medical Outcomes Partnership
OpenFHE: open-source fully homomorphic encryption
ReLU: rectified linear unit
RLWE: ring learning with errors
RNN: recurrent neural network
RNS: residue number system
RTT: round-trip time
SIMD: single instruction, multiple data
SMPC: secure multiparty computation
SRNN: sliced recurrent neural network
WAN: wide area network

## References

[1] Mitani AA, Haneuse S (2020). Small data challenges of studying rare diseases. JAMA Netw Open.

[2] Pereira T, Morgado J, Silva F (2021). Sharing biomedical data: strengthening AI development in healthcare. Health Care (Don Mills).

[3] Hussain F, Nauman M, Alghuried A, Alhudhaif A, Akhtar N (2023). Leveraging big data analytics for enhanced clinical decision-making in healthcare. IEEE Access.

[4] Yusuf S, Joseph P, Rangarajan S (2020). Modifiable risk factors, cardiovascular disease, and mortality in 155 722 individuals from 21 high-income, middle-income, and low-income countries (PURE): a prospective cohort study. Lancet.

[5] Hansen E (1997). HIPAA (Health Insurance Portability and Accountability Act) rules: federal and state enforcement. Med Interface.

[6] Scheibner J, Raisaro JL, Troncoso-Pastoriza JR (2021). Revolutionizing medical data sharing using advanced privacy-enhancing technologies: technical, legal, and ethical synthesis. J Med Internet Res.

[7] (2024). IBM report: escalating data breach disruption pushes costs to new highs. IBM Security.

[8] Mehrabi S, Sohn S, Li D (2015). 2015 International Conference on Healthcare Informatics.

[9] Choi E, Bahadori MT, Schuetz A, Stewart WF, Sun J (2016). Doctor AI: predicting clinical events via recurrent neural networks. JMLR Workshop Conf Proc.

[10] Villegas M, Gonzalez-Agirre A, Gutiérrez-Fandiño A (2023). Predicting the evolution of COVID-19 mortality risk: a recurrent neural network approach. Comput Methods Programs Biomed Update.

[11] Min X, Li W, Yang J, Xie W, Zhao D (2022). Dual-level diagnostic feature learning with recurrent neural networks for treatment sequence recommendation. J Biomed Inform.

[12] Phong LT, Aono Y, Hayashi T, Wang L, Moriai S (2018). Privacy-preserving deep learning via additively homomorphic encryption. IEEE Trans Inf Forensics Secur.

[13] Kaissis G, Ziller A, Passerat-Palmbach J (2021). End-to-end privacy preserving deep learning on multi-institutional medical imaging. Nat Mach Intell.

[14] Frigerio L, de Oliveira AS, Gomez L, Duverger P, Dhillon G, Karlsson F, Hedström K, Zúquete A (2019). 34th IFIP International Conference on ICT Systems Security and Privacy Protection.

[15] Fekri MN, Grolinger K, Mir S (2022). Distributed load forecasting using smart meter data: Federated learning with Recurrent Neural Networks. Int J Electr Power Energy Syst.

[16] Liu Y, Yu JJQ, Kang J, Niyato D, Zhang S (2020). Privacy-preserving traffic flow prediction: a federated learning approach. IEEE Internet Things J.

[17] Singh M, Madhulika M, Bansal S (2022). Computational Intelligence in Pattern Recognition CIPR 2022.

[18] Gandhi N, Mishra S, Bharti SK, Bhagat K (2021). 2021 IEEE International Conference on Electronics, Computing and Communication Technologies.

[19] Warnat-Herresthal S, Schultze H, Shastry KL (2021). Swarm learning for decentralized and confidential clinical machine learning. Nature.

[20] Nasr M, Shokri R, Houmansadr A (2019). 2019 IEEE Symposium on Security and Privacy.

[21] Mothukuri V, Parizi RM, Pouriyeh S, Huang Y, Dehghantanha A, Srivastava G (2021). A survey on security and privacy of federated learning. Future Generation Computer Systems.

[22] Kariyappa S, Guo C, Maeng K (2023). Proceedings of the 40th International Conference on Machine Learning.

[23] McMahan HB, Ramage D, Talwar K, Zhang L Learning differentially private recurrent language models. https://openreview.net/pdf?id=BJ0hF1Z0b.

[24] Blanco-Justicia A, Sánchez D, Domingo-Ferrer J, Muralidhar K (2023). A critical review on the use (and misuse) of differential privacy in machine learning. ACM Comput Surv.

[25] Yue K, Jin R, Wong CW, Baron D, Dai H (2023). 32nd USENIX Security Symposium.

[26] Yang Y, Gohari P, Topcu U (2023). On the privacy risks of deploying recurrent neural networks in machine learning models. Proc Priv Enhanc Technol.

[27] Jayaraman B, Evans D (2019). 28th USENIX Security Symposium.

[28] Rathee D, Rathee M, Goli RKK (2021). 2021 IEEE Symposium on Security and Privacy.

[29] Feng Q, He D, Liu Z, Wang H, Choo KKR (2020). SecureNLP: a system for multi-party privacy-preserving natural language processing. IEEE Trans Inf Forensics Secur.

[30] Podschwadt R, Takabi D (2021). 2021 IEEE 14th International Conference on Cloud Computing.

[31] Tosevski V, Gulak G (2025). 2025 IEEE International Conference on Acoustics, Speech and Signal Processing (ICASSP 2025).

[32] Wang Z, Ikeda M (2023). 2023 International Joint Conference on Neural Networks (IJCNN).

[33] Jang J, Lee Y, Kim A (2022). Proceedings of the 2022 ACM on Asia Conference on Computer and Communications Security.

[34] Xie Q, Guo H, Wang W (2025). Proceedings of the 34th International Joint Conference on Artificial Intelligence.

[35] Paul J, Annamalai M, Ming W, Badawi AA, Veeravalli B, Aung KMM (2021). Privacy-preserving collective learning with homomorphic encryption. IEEE Access.

[36] Sav S, Diaa A, Pyrgelis A, Bossuat JP, Hubaux JP (2023). Privacy-preserving federated recurrent neural networks. Proc Priv Enhanc Technol.

[37] Guo Q, Nabokov D, Suvanto E, Johansson T (2024). 33rd USENIX Security Symposium.

[38] Bergamaschi F, Costache A, Dachman-Soled D (2025). 28th IACR International Conference on Practice and Theory of Public-Key Cryptography.

[39] Lu Y, Tian Y, Zhou T, Zhu S, Li J (2021). Multicenter privacy-preserving Cox analysis based on homomorphic encryption. IEEE J Biomed Health Inform.

[40] Li B, Micciancio D, Schultz-Wu M, Sorrell J (2022). 42nd Annual International Cryptology Conference.

[41] Paverd A, Martin A, Brown I (2014). Modelling and automatically analysing privacy properties for honest-but-curious adversaries. https://ajpaverd.org/publications/casper-privacy-report.pdf.

[42] Yu Z, Liu G Sliced recurrent neural networks. https://aclanthology.org/C18-1250.pdf.

[43] Johnson AEW, Pollard TJ, Shen L (2016). MIMIC-III, a freely accessible critical care database. Sci Data.

[44] Goldberger AL, Amaral LAN, Glass L (2000). PhysioBank, PhysioToolkit, and PhysioNet. Circulation.

[45] Reyna MA, Josef CS, Jeter R (2020). Early prediction of sepsis from clinical data: the PhysioNet/Computing in Cardiology Challenge 2019. Crit Care Med.

[46] Hripcsak G, Duke JD, Shah NH (2015). Observational health data sciences and informatics (OHDSI): opportunities for observational researchers. Stud Health Technol Inform.

[47] Cheon JH, Choe H, Passelègue A, Stehlé D, Suvanto E (2024). Proceedings of the 2024 ACM SIGSAC Conference on Computer and Communications Security.

[48] Cheon JH, Kim A, Kim M, Song Y (2017). 23rd International Conference on the Theory and Applications of Cryptology and Information Security.

[49] Akleylek S, Dağdelen Ö, Yüce Tok Z (2016). 2nd International Conference on Cryptography and Information Security in the Balkans.

[50] Bajard JC, Eynard J, Hasan MA, Zucca V (2017). 23rd International Conference on Selected Areas in Cryptography.

[51] Smart NP, Vercauteren F (2014). Fully homomorphic SIMD operations. Des Codes Cryptogr.

[52] Kim A, Papadimitriou A, Polyakov Y (2022). Cryptographers’ Track at the RSA Conference 2022.

[53] Jiang X, Kim M, Lauter K, Song Y (2018). Proceedings of the 2018 ACM SIGSAC Conference on Computer and Communications Security.

[54] Rizomiliotis P, Triakosia A (2022). Proceedings of the 2022 on Cloud Computing Security Workshop.

[55] Albrecht M, Chase M, Chen H (2018). Homomorphic encryption standard. http://homomorphicencryption.org/wp-content/uploads/2018/11/HomomorphicEncryptionStandardv1.1.pdf.

[56] (2023). Microsoft SEAL (release 4.1). Microsoft Research.

[57] Cascarano A, Mur-Petit J, Hernández-González J (2023). Machine and deep learning for longitudinal biomedical data: a review of methods and applications. Artif Intell Rev.

[58] Mouchet C, Troncoso-Pastoriza J, Bossuat JP, Hubaux JP (2021). Multiparty homomorphic encryption from ring-learning-with-errors. Proc Priv Enhanc Technol.

[59] Koirala N, Takeshita J, Stevens J, Jung T (2024). Summation-based private segmented membership test from threshold-fully homomorphic encryption. Proc Priv Enhanc Technol.

[60] Alexandru A, Badawi AA, Micciancio D, Polyakov Y (2026). Application-aware approximate homomorphic encryption: configuring FHE for practical use. IACR Commun Cryptol.

[61] Jiang S, Yang H, Xie Q (2025). Towards compute-efficient byzantine-robust federated learning with fully homomorphic encryption. Nat Mach Intell.

[62] Xie Q, Jiang S, Jiang L (2024). Efficiency optimization techniques in privacy-preserving federated learning with homomorphic encryption: a brief survey. IEEE Internet Things J.

[63] Papernot N, Steinke T Hyperparameter tuning with Renyi differential privacy. https://openreview.net/pdf?id=-70L8lpp9DF.

[64] Kuo K, Thaker P, Khodak M (2023). On noisy evaluation in federated hyperparameter tuning. Proc Mach Learn Syst.

[65] Khodak M, Tu R, Li T (2021). Federated hyperparameter tuning: challenges, baselines, and connections to weight-sharing. Adv Neural Inf Process Syst.

[66] Cheon JH, Kim W, Park JH (2022). Efficient homomorphic evaluation on large intervals. IEEE Trans Inf Forensics Secur.

[67] Silva I, Moody G, Mark R, Celi LA (2012). Predicting mortality of ICU patients: the PhysioNet/Computing in Cardiology Challenge 2012 (version 1.0.0). https://physionet.org/content/challenge-2019/1.0.0/.

[68] Reyna M, Josef C, Jeter R (2019). Early prediction of sepsis from clinical data: the PhysioNet/Computing in Cardiology Challenge 2019 (version 1.0.0).

[69] Johnson A, Pollard T, Mark R (2016). MIMIC-III Clinical Database (version 1.4).

